# Sex differences in adaptation to intermittent post-exercise sauna bathing in trained middle-distance runners

**DOI:** 10.1186/s40798-021-00342-6

**Published:** 2021-07-23

**Authors:** Nathalie V. Kirby, Samuel J. E. Lucas, Thomas G. Cable, Oliver J. Armstrong, Samuel R. Weaver, Rebekah A. I. Lucas

**Affiliations:** 1 School of Sport Exercise and Rehabilitation Sciences, University of Birmingham, Birmingham, UK; 2 Human and Environmental Physiology Research Unit, University of Ottawa, Ottawa, K1N 6N5 Canada; 3 Loughborough University, Loughborough, UK; 4 Performance Centre, University of Birmingham Sport, Birmingham, UK

## Abstract

**Background:**

The purpose of this study was to investigate the effect of sex on the efficacy of intermittent post-exercise sauna bathing to induce heat acclimation and improve markers of temperate exercise performance in trained athletes.

**Methods:**

Twenty-six trained runners (16 female; mean ± SD, age 19 ± 1 years, V̇O_2max_ F: 52.6 ± 6.9 mL⋅kg^−1^⋅min^−1^, M: 64.6 ± 2.4 mL⋅kg^−1^⋅min^−1^) performed a running heat tolerance test (30 min, 9 km⋅h^−1^/2% gradient, 40 °C/40%RH; HTT) and temperate (18 °C) exercise tests (maximal aerobic capacity [V̇O_2max_] and lactate profile) pre and post 3 weeks of normal exercise training plus 29 ± 1 min post-exercise sauna bathing (101–108 °C) 3 ± 1 times per week.

**Results:**

Females and males exhibited similar reductions (interactions *p *> 0.05) in peak rectal temperature (− 0.3 °C; *p *< 0.001), skin temperature (− 0.9 °C; *p* < 0.001) and heart rate (− 9 beats·min^−1^; *p* = 0.001) during the HTT at post- vs pre-intervention. Only females exhibited an increase in active sweat glands on the forearm (measured via modified iodine technique; F: + 57%, *p* < 0.001; M: + 1%, *p* = 0.47). Conversely, only males increased forearm blood flow (measured via venous occlusion plethysmography; F: + 31%, *p* = 0.61; M: + 123%; *p* < 0.001). Females and males showed similar (interactions *p* > 0.05) improvements in V̇O_2max_ (+ 5%; *p* = 0.02) and running speed at 4 mmol·L^−1^ blood lactate concentration (+ 0.4 km·h^−1^; *p* = 0.001).

**Conclusions:**

Three weeks of post-exercise sauna bathing effectively induces heat acclimation in females and males, though possibly amid different thermoeffector adaptations. Post-exercise sauna bathing is also an effective ergogenic aid for both sexes.

**Supplementary Information:**

The online version contains supplementary material available at 10.1186/s40798-021-00342-6.

## Key points

- Females may require longer duration exercise-heat acclimation protocols or a greater heat stimulus to induce heat acclimation. Although post-exercise passive heating is emerging as a viable alternative to exercise-heat acclimation, with evidence of additional benefits for temperate exercise performance, the effect of sex on the efficacy of post-exercise heating is unknown.
- Three weeks of post-exercise sauna bathing effectively induced heat acclimation in females and males, though possibly amid different thermoeffector adaptations (i.e. only males increased forearm blood flow and only females increased forearm sweat gland activity).
- Post-exercise sauna bathing was similarly effective for both sexes in improving maximal and submaximal exercise performance markers in temperate conditions.

## Introduction

Hot ambient temperatures adversely impact endurance performance [1]. Athletes can use repeated bouts of heat stress (i.e. heat acclimation) to improve exercise performance in the heat, as heat acclimation induces a myriad of integrative adaptations that improve cardiovascular and thermoeffector function and lowers skin and core body temperatures [2]. There is growing interest in using passive forms of heat acclimation to drive these favourable physiological adaptations, on account of their practical and accessible nature [3]. Specifically, post-exercise passive heating (e.g. post-exercise sauna bathing) is recommended [4, 5], as athletes begin passive heating bouts at elevated body temperatures (a key driver of heat acclimation) due to the preceding exercise bout [6]. Indeed, data from our laboratory [7] and the literature [8, 9] demonstrate that repeated bouts of post-exercise sauna bathing elicit heat acclimation adaptations.

A post-exercise sauna bathing protocol may elicit different acute and/or adaptive responses in females compared to males for a number of reasons. Acutely, females have been observed to exhibit a higher heart rate despite a similar rise in (sublingual) temperature to males during 30-min dry sauna exposure independent of exercise [10]. Females also exhibit reduced sudomotor sensitivity [11] and a lower sweat rate overall [12] when exposed to dry heat. It stands to reason that the extreme heat experienced in a dry sauna (typically > 80 °C), where ambient temperatures far exceed skin temperatures, could pose an additional thermoregulatory challenge for females because of their greater surface area-to-mass ratio and consequently greater dry heat gain [13]. Moreover, females exhibit even greater elevations in post-exercise sweating and vasodilation onset thresholds than males [14, 15]. Thus, when severe heat stress is superimposed onto exercise recovery, such as during post-exercise sauna bathing, females may experience comparatively greater thermoregulatory strain. The first aim of the current study was to investigate the effects of sex on the acute response to post-exercise sauna bathing.

Despite possibly inducing greater thermoregulatory strain in females, a post-exercise sauna bathing intervention may not be as effective for inducing adaptation in females as compared to males. Females have been observed to demonstrate a slower adaptive response to heat acclimation as compared to males [16], even when females experience comparatively greater thermoregulatory and cardiovascular strain during the initial heat exposures [17]. Thus, 3 weeks of intermittent post-exercise sauna bathing may be more effective for inducing heat acclimation in males than in females. The second aim of the current study was to investigate the effects of sex on the adaptive response to repeated intermittent bouts of post-exercise sauna bathing.

Though there is conflicting evidence as to whether the physiological adaptations attained through heat acclimation improve exercise performance in cool or temperate conditions [18, 19], studies investigating active heat acclimation’s ergogenic potential have not directly compared its efficacy in both sexes. Moreover, studies investigating the ergogenic effects of post-exercise hot water immersion [20] as well as sauna bathing post [8] and independent of exercise [21] have been either in male or mixed-sex [7] cohorts, where sex-specific responses were not disaggregated. Considering the aforementioned potential sex differences in adaptive response to heat stress and females’ comparatively reduced aerobic capacity trainability [22], comparing the ergogenic potential of post-exercise sauna bathing between sexes is warranted. Therefore, the third aim of this study was to investigate the effect of sex on markers of temperate exercise performance following repeated bouts of intermittent post-exercise sauna bathing.

The overall purpose of this study was to investigate the effect of sex on the efficacy of intermittent post-exercise sauna bathing to induce heat acclimation and improve markers of maximal and submaximal exercise performance (lactate profile, maximal aerobic capacity and time-to-exhaustion) in temperate conditions in trained athletes. We hypothesised that an acute bout of post-exercise sauna bathing would result in greater thermoregulatory strain in females. However, despite this greater thermoregulatory strain, it was further hypothesised that repeated post-exercise sauna bathing would be more effective in males for inducing desirable adaptations during both exercise-heat stress and temperate exercise, in accordance with previous literature [16, 17, 22].

## Methods

### Ethical approval

This study was approved by the University of Birmingham Ethics Committee (ERN_18-0958) and conformed to the standards set by the Declaration of Helsinki (2013). All participants were informed of the experimental procedures and possible risks involved in the study before providing written consent.

### General overview and design

This study expands on previous findings from our laboratory [7] by substantially increasing the sample size to allow for sex comparison and assessing additional outcome measures. As such, some participants’ data included in these analyses have been previously published [7].

**Fig. 1:**
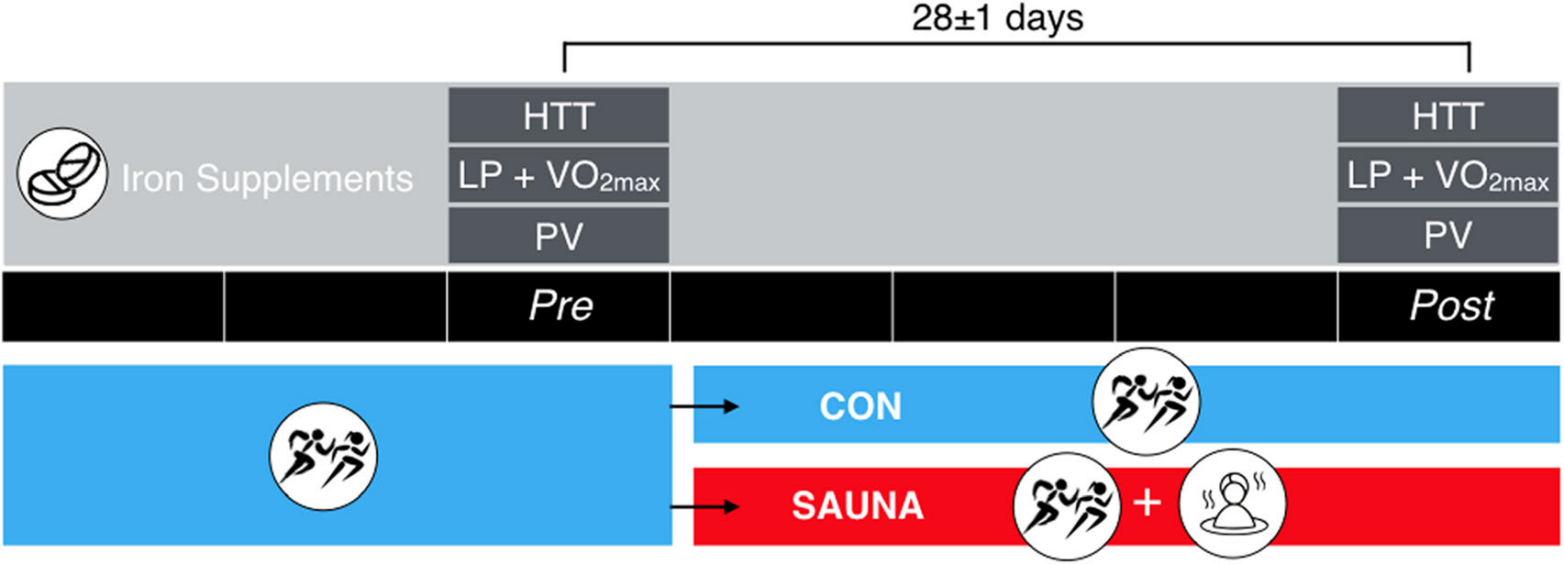
Schematic of the experimental design. Females and males in the intermittent post-exercise sauna bathing intervention group (SAUNA) and control group (CON) completed temperate exercise tests (18 °C) consisting of lactate profile (LP) and maximal aerobic capacity (V̇O_2max_) tests, a running heat tolerance test (HTT; 30 min, 9 km·h^−1^/2% gradient, 40 °C/40%RH), and a plasma volume assessment at baseline (Pre) and following three weeks (Post) of sauna intervention or control. Respective tests were completed ~ 28 days apart. Black bars indicate weeks

The general overview and study design matches the 3-week intervention described in Kirby et al. [7]. A battery of experimental trials was completed within a 1-week timeframe at baseline (Pre) and following 3 weeks (Post) of either normal endurance training (CON) or normal endurance training with the post-exercise sauna bathing intervention (SAUNA). Experimental trials included a running heat tolerance test (HTT), a temperate exercise test (consisting of a lactate profile test and test of maximal aerobic capacity [V̇O_2max_]) and a morning resting heart rate and finger-prick blood sample (Fig. 1).

Each participant completed a general health questionnaire and female participants completed a menstrual cycle questionnaire. Throughout the protocol, participants recorded their training (type, distance, frequency and session perceived exertion) and female participants recorded their menstrual cycle. All participants took daily iron tablets (65 mg ferrous sulphate; Nature Made, West Hills, CA, USA) from 2 weeks prior to the first experimental trial until the completion of the protocol. Since (1) iron deficiency is common in highly trained endurance athletes, particularly female endurance athletes [23], and (2) iron deficiency is known to impair adaptive responses to exercise training [24], iron supplements were intended to ensure equal efficacy of normal training alone in both sexes.

Experimental trials were arranged ~ 28 days apart to test female participants during the same menstrual cycle phase, as per a typical menstrual cycle. Participants self-allocated into groups prior to baseline testing. This experiment was conducted in the UK between the months of October and March to minimise any natural heat acclimatisation.

### Participants

Eighty trained middle-distance and cross-country runners were recruited from the university’s athletics club to participate in this study. However, due to various reasons (injury, scheduling etc.), only 41 athletes completed experimental trials post-intervention. Participant characteristics are summarised in Table 1. Of these 41 athletes, 1 female CON participant did not complete the temperate exercise tests post-intervention and 3 SAUNA (F: *n* = 2; M: *n* = 1) and 2 CON (F: *n* = 1; M: *n* = 1) participants did not complete the HTT post-intervention.

**Table 1:** Participants’ characteristics. Data are presented as mean ± SD

	SAUNA		CON	
	*Females*	*Males*	*Females*	*Males*
Participants (*n*)	16	10	7	8
Age (years)	19 ± 1	20 ± 1	19 ± 2	20 ± 2
Height (cm)	166 ± 7^#^	177 ± 6	166 ± 8^#^	178 ± 7
Body mass (kg)	54 ± 9^#^	66 ± 7	56 ± 7^#^	64 ± 6
BSA (m^2^)	1.59 ± 0.11^#^	1.82 ± 0.13	1.62 ± 0.14^#^	1.80 ± 0.09
BSA-mass ratio (cm^2^·kg^−1^)	298 ± 28^#^	275 ± 15	289 ± 13	283 ± 16
V̇O_2max_ (mL·kg^−1^·min^−1^)	52.6 ± 6.9^#^	64.6 ± 2.4	52.9 ± 4.2^#^	63.6 ± 3.2

*SAUNA* post-exercise sauna bathing intervention group, *CON*, control group, *BSA* body surface area, *V̇O*_*2max*_ maximal aerobic capacity

^#^Significantly different from males within groups (*p* < 0.05)

A subset of SAUNA group participants was sampled at the first post-exercise sauna bathing session to quantify the acute physiological response to post-exercise sauna bathing (F: age 19 ± 1 years, body mass 54.4 ± 6.7 kg, height 167 ± 7 cm, body surface area 1.61 ± 0.10 m^2^, body surface area-to-mass ratio 297 ± 21 cm^2^·kg^−1^, V̇O_2max_ 52.0 ± 7.2 mL·kg^−1^·min^−1^, *n* = 7; M: age 20±1 years, body mass 66.4 ± 8.6 kg, height 177 ± 7 cm, body surface area 1.82 ± 0.15 m^2^, body surface area-to-mass ratio 276 ± 16 cm^2^·kg^−1^, V̇O_2max_ 64.3 ± 1.9 mL·kg^−1^·min^−1^, *n* = 8). In this subset, females were shorter and lighter, and had a lower body surface area, body surface area-to-mass ratio and V̇O_2max_ than males (all *p*<0.05). Females and males were of a similar age (*p* = 0.14).

Female participants included in the SAUNA and CON groups were eumenorrheic (regular menstrual cycles lasting 25–36 days; *n* = 13), oligomenorrheic (menstrual cycles > 40 days; *n* = 2), amenorrheic (> 3 months without menstruation; *n* = 2), or using various forms of hormonal contraceptives (monophasic combined oral contraceptive pill, *n* = 2; progestin-only oral contraceptive pill, *n* = 1; implant, *n* = 1; contraceptive coil, *n* = 2). Female participants completed all Pre and Post experimental trials 28±2 days apart. Eleven of the eumenhorreic female participants completed HTTs in the same phase of their menstrual cycle and nine completed temperate exercise tests in the same phase. The remainder completed Pre and Post tests in the following phases, HTTs: follicular then luteal (SAUNA, *n* = 1), luteal then follicular (SAUNA, *n* = 1); temperate exercise tests: follicular then luteal (SAUNA, *n* = 1; CON, *n* = 1), luteal then follicular (SAUNA, *n* = 2). Participants using oral contraceptives (*n* = 3) completed all tests in the active pill-taking phase. The subset of female participants sampled at the first post-exercise sauna bathing session were eumenorrheic (follicular phase, *n* = 3; luteal phase, *n* = 1), amenorrheic (*n* = 1) or using a contraceptive coil (*n* = 2). Female participants reported that they did not experience any negative menstrual or pre-menstrual symptoms that may have affected performance, or otherwise were not scheduled for experimental tests near menstruation.

### Post-exercise sauna bathing

In the subset of participants sampled in greater detail at the first post-exercise sauna session (*n* = 15), participants ended their outdoor run (7–11 °C, 85–93%RH) at the University of Birmingham’s Sport and Fitness Centre between 19:00 and 20:00 h. Participants entered the sauna 17 ± 12 min after their run, following measurement of nude body mass and instrumentation. Rectal temperature (T_rec_) was recorded immediately before entering the sauna. Participants were asked to remain in the sauna for 30 min or until they could no longer tolerate the discomfort, as per previous investigations [8, 9]. The dry (5–9%RH) sauna was ~ 104 °C at 1.8m, ~ 86 °C at participants’ approximate chest height and ~ 60 °C at seat height (iButton Hygrochron Logger, Maxim Integrated, California, USA). In the sauna, participants sat upright and were allowed to drink water ad libitum (the amount of which was recorded). Heart rate (HR) and T_rec_ were recorded during the final minute of exposure. Nude body mass was measured approximately 5–10 min following sauna exposure.

For the remaining post-exercise sauna sessions, where participants were not instrumented (i.e. in all SAUNA participants), participants entered the sauna within ~ 5 min of finishing outdoor exercise. Sauna bathing typically followed low-intensity, continuous exercise training sessions (i.e. “easy runs” or “long runs”). Participants drank ad libitum and only HR was recorded during the final minute of exposure.

### Experimental trials

Participants performed all experimental trials at the same time of day (± 2 h). Participants were asked not to exercise before visiting the laboratory on the day of the experimental trials, to abstain from caffeine for 4 h before trials, and not to consume alcohol on the day prior to any experimental trials. Participants were not taking any regular medications (besides those taking hormonal contraceptive pills). Participants were instructed in advance to eat a light and nutritious meal and to stay well-hydrated, and informed that they would be asked to recall the food they had eaten prior to experimental tests and to repeat the same diet when re-tested. Participants voided their bladder upon arrival to the laboratory to provide a urine sample, which was analysed for osmolality. If urine osmolality was ≥ 700 mOsm·kg^−1^ (Osmocheck, Vitech Scientific Ltd., West Sussex, UK) [25], participants drank 250 mL of water and did not begin exercising for at least 20 min.

#### Running heat tolerance test

The running heat tolerance test (HTT) was performed in hot conditions (40 °C, 40%RH) in an environmental chamber (TIS Services, Hampshire, UK) with a fan-generated airflow of ∼ 4 m·s^−1^ (MIW Office Solutions, County Durham, UK), as described by Mee et al. [26]. Participants ran on a treadmill (H/P/Cosmos Quasar 4.0, H/P/Cosmos, Germany) at 9 km·h^−1^ and 2% gradient for 30 min [26]. Rectal temperature (T_rec_), skin temperature (T_sk_) and HR were measured continuously. Ratings of perceived exertion (RPE_6–20_), thermal comfort and thermal sensation were obtained in the final minute of the HTT. Sweat gland activity of the forearm and upper back were measured immediately following the HTT (within 2–10 s). Participants then sat in a chair with their left arm supported at heart level, and forearm blood flow (FBF) was recorded at 4:26 ± 1:42 min post-exercise. FBF measurement times post-exercise differed by 26 ± 45 s within-participant, between tests at Pre and Post. Towel-dried, nude body mass was recorded to 0.1 kg using digital scales (Seca 877, Seca, Hamburg, Germany) before and immediately after the HTT to estimate whole-body sweat loss. Participants wore socks, shoes, shorts and, in females, a sports bra. Two CON males opted to wear a t-shirt and one CON male opted to wear a sleeveless running shirt for both Pre and Post tests. Drinking was not permitted during the HTT.

#### Lactate profile test

The lactate profile test was performed in temperate conditions (~ 18 °C) with a fan-generated airflow of ∼ 3 m·s^−1^. After completing a light 10-min warm-up, participants completed a step-style incremental treadmill test with 3-min stages and 30-s stops between stages for measurement of blood lactate concentration ([La^−^]). The test began at 1% gradient [27] and a speed 4 km·h^−1^ slower than a recent 5-km race-pace, increasing 1 km·h^−1^ at each stage thereafter. HR and respiratory gas exchange were continuously measured. The test was terminated when [La^−^] exceeded 4 mmol·L^−1^, which occurred following 4–7 stages. The facemask was removed and the fan was switched off immediately. Participants were then seated on a chair placed on the treadmill at 30 s from the cessation of exercise, and sat quietly until 3 min post-exercise, whilst HR recovery was measured continuously.

#### Maximal aerobic capacity test

Approximately 10 min after completing the lactate profile test, participants performed a ramp-style V̇O_2max_ test. The test began at 1% gradient and at a speed 2 km·h^−1^ slower than the speed at which participants exceeded 4 mmol·L^−1^ [La^−^]. Speed increased 1 km·h^−1^ each minute for the first two stages, after which the gradient was increased by 1% each minute until volitional exhaustion. Participants were given consistent and loud encouragement during the test. When participants were re-tested post-intervention, the starting speed and progression of the protocol were repeated to allow for measurement of time-to-exhaustion (TTE). HR and respiratory gas exchange were continuously measured. Capillary [La^−^] was measured 5 min from the time of exhaustion.

#### Resting heart rate and plasma volume visit

On a separate visit (between 9:00 and 11:00 h), participants rested in a supine position for a minimum of 10 min, at which time resting HR and haemoglobin (Hb) and haematocrit (Hct) were assessed for measurement of plasma volume changes.

### Measures

#### Body temperatures

T_rec_ was measured using a rectal thermistor inserted 10 cm past the anal sphincter (Mon-a-Therm, Covidien, Mansfield, MA, USA). Peak T_rec_ was calculated as the average T_rec_ in the final minute of the HTT. T_recRISE_ was calculated as the change in T_rec_ from 0 to 30 min of the HTT. Skin temperatures were recorded using skin thermistors (Squirrel Thermal Couples, Grant Instruments, Cambridge, UK) attached to four sites on the left side of the body: pectoralis major, biceps brachii, rectus femoris and gastrocnemius lateral head. T_sk_ was calculated as a weighted average according to Ramanathan [28]. Skin and rectal temperatures were continuously logged at 30-s intervals (Squirrel 2020 series, Eltek, Ltd., UK). If data from an individual T_sk_ site was lost, the missing data were modelled according to the slope/pattern of other data points from that participant during the previous test.

#### Cardiovascular

HR (Polar Electro, Kempele, Finland) was recorded continuously (sampling rate of 1 Hz) on the Polar Beat application (Polar Beat, Kempele, Finland). Peak HR during the HTT was calculated as the average HR in the final minute. Submaximal HR data during the lactate profile test were calculated as the average of the final minute of the stage at which participants exceeded 4 mmol·L^−1^ [La^−^]. HR recovery from submaximal temperate exercise was calculated at four intervals; the 10-s average HR following the immediate cessation of exercise, and the 10-s preceding 1-, 2- and 3-min post-exercise. FBF was quantified using venous occlusion plethysmography [29], with a strain gauge on the widest part of the forearm (position measured from the elbow to replicate strain gauge position across trials) supported at heart level. Blood flow to the hand was occluded using a wrist cuff at a pressure > 220 mmHg to prevent the inclusion of hand circulation in the calculations of FBF. An upper arm cuff was occluded at a pressure of 45 mmHg for 10 s, and voltage output was recorded (PowerLab, ADInstruments, Dunedin, New Zealand). Outputs of 4–8 s post-upper arm occlusion were analysed offline (Labchart; ADInstruments) for maximal flow response, as detailed by Wythe and colleagues [30]. FBF was normalised to forearm volume, as calculated using water displacement. FBF was not recorded in two SAUNA (F: *n* = 1; M: *n* = 1) and two CON (M: *n* = 2) participants due to technical difficulties.

#### Sweating

Estimated sweat loss was calculated as the difference between pre- and post-HTT nude body mass, and normalised as a percentage of body mass. Active sweat glands were quantified using a modified-iodine paper technique with blinded computer-aided analysis [31]. Samples were collected by the same researcher (NVK) from the dorsal side of the thickest segment of the forearm and mid-scapula on the upper back. Upper back sweat gland activity was not recorded in the two male CON participants who wore a t-shirt during the HTTs.

#### Cardiopulmonary

Respiratory gas exchange was sampled breath-by-breath using open-circuit spirometry (Vyntus CPX, Jaeger, Wuerzberg, Germany). Respiratory gas exchange data were exported as 5-s values and used to calculate running economy and respiratory exchange ratio (RER) during the lactate profile test, as well as maximal absolute (L·min^−1^) and relative (mL·kg^−1^·min^−1^) oxygen consumption during V̇O_2max_ tests. Running economy (mL·kg^−1^·km^−1^) was calculated as oxygen consumption (mL·kg^−1^·min^−1^) divided by treadmill speed (km·h^−1^) [32]. Submaximal RER and running economy data were calculated as the average of the final minute of the stage at which participants exceeded 4 mmol·L^−1^ [La^−^]. V̇O_2max_ was calculated as the highest rolling 30-s average attained during the test. Successful attainment of V̇O_2max_ required meeting two of the following three criteria: 1) [La^−^] ≥ 8 mmol·L^−1^ [33], 2) RER ≥ 1.10 [34], 3) maximal HR ≥ 90% of age-predicted maximal HR (220−age). Additionally, a plateau was confirmed both visually and systematically by a 5-s average value ≥ 2 standard deviations lower than the linearly predicted V̇O_2_ increase.

#### Blood sampling and analysis

Changes in resting plasma volume were determined using a finger prick capillary sample for the immediate measurement of Hb and Hct via automated blood analyser (i-STAT, Abbott, NJ, USA). Plasma volume was calculated according to the methodology outlined by Dill & Costill [35]. Plasma volume was measured in 18 SAUNA (F: *n* = 9; M: *n* = 9) and 10 CON (F: *n* = 4; M: *n* = 6) participants due to technical difficulties, issues with scheduling or aversion to blood. Blood lactate measures were taken from a fingertip capillary sample and immediately analysed using a Biosen C-Line Lactate analyser (EKF Diagnostics, Penarth, UK), which was quality checked each day and calibrated every 60 min. Running speed at 4 mmol·L^−1^ [La^−^] was determined using a custom Matlab script (Mathworks Inc, Natick, USA) to fit a third-order polynomial curve to each individual dataset and interpolate the running speed at 4 mmol·L^−1^ [La^−^].

#### Perceptual

RPE for training sessions was rated on a 1–10-point scale (RPE_1–10_), and RPE for experimental trials was measured using the 6–20-point Borg Scale (RPE_6–20_) [36]. Thermal sensation and thermal comfort were measured using modified 13- and 10-point scales, respectively [37].

### Statistical analysis

All data were analysed using SPSS statistical software (SPSS version 25.0.0, SPSS, Chicago, IL, USA). Data collected during the first post-exercise sauna session were compared between-sex using independent *t*-tests.

We have previously demonstrated the SAUNA intervention to be superior to normal training (i.e., CON) for both heat acclimation indices and temperate exercise performance markers in a subset of participants [7], and confirmed these findings in the current, larger dataset using a two-way (*group × sex*) analysis of covariance (ANCOVA), with change scores (∆; difference from Pre to Post) as the dependent variable and baseline absolute data as a covariate. These results are presented in Supplementary Materials. Due to high levels of dropout, the current dataset was not powered (ascertained using data from our previous study [7] with power set at β ≥ 0.80 and α = 0.05; G*Power version 3.1.9.3, Germany) for a formal three-way (*time × group × sex*) analysis of variance (ANOVA) comparison. Therefore, we have investigated the effects of sex on post-exercise sauna bathing responses by examining and presenting results from data from the SAUNA group only. Specifically, the effect of sex on physiological and perceptual data at Pre and Post were compared within the SAUNA group using a two-way (*time × sex*) ANOVA. A power analysis (power β ≥ 0.80, α = 0.05) using effect sizes from data in our previous study [7] indicated that *n* = 14 participants was a sufficient sample size to detect any favourable changes in main outcome measures (i.e. V̇O_2max_, speed at 4 mmol·L^−1^ [La^−^] and peak T_rec_ and HR) as a result of normal training plus post-exercise sauna bathing. Any discrepancies between the results of the between-group ANCOVA analyses and the analyses described below are noted within the relevant “Results” sections, and where not stated, should be assumed to be in agreement. HR recovery at each post-exercise 1-min interval was compared using a 3-way (*time × sex × interval*) ANOVA. Average weekly training frequency and running distance were compared between sexes using independent *t*-tests. Average weekly frequency and perceived exertion of each type of training session (i.e. easy runs, tempo sessions, high-intensity sessions and long runs) were compared using a two-way (*type × sex*) ANOVA.

Normality of the data was confirmed using the Shapiro–Wilk test. Sphericity of the data was assessed using Mauchly’s test of sphericity, and Greenhouse–Geisser corrections were applied where assumptions of sphericity were violated. When a significant main effect was found, a Dunn–Bonferroni-corrected post hoc analysis was used to compare pairwise differences. Significance was set at *p* ≤ 0.05. Absolute data are presented as mean ± standard deviation (SD), and between-sex and pre- to post-intervention mean differences are presented with corresponding 95% confidence intervals [lower limit, upper limit].

## Results

### Training

In the weeks between pre- and post-intervention testing, the average weekly running distance in the SAUNA group was 26.7 km [10.9, 42.6] greater in males (69.5±13.0 km) than in females (42.8±17.1 km; *sex*: *p* < 0.001). There were no sex or group differences in training session frequency, type or the associated perceived exertion (all *p* > 0.05; Supplementary Material).

### Post-exercise sauna bathing intervention

Responses during the first post-exercise sauna session and the preceding exercise bout are detailed in Table 2. Immediately before the first post-exercise sauna bathing session, the subset of female and male participants sampled ran for a similar duration outdoors (mean difference between sexes [95% CI]: 12 min [-3, 27]; *p* = 0.09), though females exhibited a higher mean HR during the run (25 beats·min^−1^ [11, 40]; *p *< 0.01). Female and male participants exhibited a similar T_rec_ upon entering the sauna (0.0 °C [− 0.4, 0.4]; *p* = 0.96) and reached a similar T_rec_ when they exited the sauna (0.2 °C [− 0.2, 0.6]; *p* = 0.23). Female and male participants exhibited a similar peak HR in the sauna (4 beats·min^−1^ [− 22, 29]; *p* = 0.76), lost a similar percentage of their body mass due to sweating (0.0% [− 0.7, 0.8]; *p* = 0.94), drank a similar amount of water (46 mL [− 237, 328]; *p* = 0.73) and therefore replaced a similar percentage of fluid lost (2% [− 125, 129]; *p* = 0.98).

**Table 2:** Stimulus duration and physiological responses to the post-exercise sauna bathing intervention. Data are presented as mean ± SD

	*Females*	*Males*
*First post-exercise sauna session*		
Exercise duration (min)	42 ± 12	55 ± 9
Mean exercising HR (beats·min^−1^)	168 ± 12^#^	143 ± 8
T_rec_ entering sauna (°C)	37.6 ± 0.4	37.6 ± 0.2
Peak T_rec_ in sauna (°C)	38.4 ± 0.5	38.6 ± 0.2
Peak HR in sauna (beats·min-^1^)	133 ± 21	131 ± 18
Sweat loss in sauna (°C)	1.2 ± 0.2	1.2 ± 0.6
Fluid consumption (mL)	886 ± 260	931 ± 237
Fluid replaced (%BM lost)	172 ± 67	158 ± 106
Sauna session duration (min)	29 ± 3	29 ± 3
*Post-exercise sauna intervention*		
Peak HR in sauna (beats·min-^1)^	123 ± 10	123 ± 8
Mean sauna session duration (min)	29 ± 1	29 ± 1
Total sauna exposure (min)	272 ± 43	277 ± 39

*BM* body mass, *HR* heart rate, *T*_*rec*_ rectal temperature

^#^Significantly different from males (*p* < 0.05)

For the remainder of the study, all females and males included in the post-exercise sauna bathing intervention attended a similar (*p* = 0.59) amount of sauna sessions (3 ± 1 sessions·week^−1^), accumulating 9 ± 1 sauna sessions before completing temperate exercise tests at Post, and 10±1 sauna sessions before completing the HTT at Post. Participants remained in the sauna for 29±1 min each session, totalling 275 ± 40 min of sauna exposure, which did not differ by sex (*p* = 0.40 and *p* = 0.68, respectively). Across all sauna sessions, peak HR was similar between female and male participants (1 beat·min^−1^ [− 7, 8]; *p* = 0.92; Table 2).

### Resting measures

Resting measures for the SAUNA group are detailed in Table 3. Females and males exhibited similar (*sex*: *p* = 0.73; *time × sex: p* = 0.49) reductions in resting T_rec_ (mean difference from Pre to Post [95% CI]: -0.2 °C [-0.1, -0.3], *time*: *p *< 0.01). Females and males also exhibited similar (*sex*: *p* = 0.30; *time × sex: p* = 0.58) reductions in resting HR (− 3 beats·min^−1^ [− 2, − 5], *time*: *p* = 0.001), although the ANCOVA comparison with the CON group showed that the reduction in resting HR was not superior to that from normal training alone (Supplementary Material). Plasma volume was 6% higher in females than in males (*sex*: *p* < 0.01), though plasma volume was not affected by post-exercise sauna bathing (− 1% [2, − 3], *time*: *p* = 0.65; *time × sex*: *p* = 0.49). Hb concentration was also greater in males than in females (1.7 g·dL^−1^ [1.2, 2.2], *sex*: *p* = 0.49), but was not affected by post-exercise sauna bathing (− 0.1 g·dL^−1^ [0.2, − 0.4], *time*: *p* = 0.56; *time × sex*: *p* = 0.48).

**Table 3:** Physiological responses at rest and during the running heat tolerance test before (Pre) and after (Post) 3 weeks of intermittent post-exercise sauna bathing. Data are presented as mean ± SD

	Females		Males	
	*Pre*	*Post*	*Pre*	*Post*
*Rest*				
Resting T_rec_ (°C)	37.2 ± 0.1	37.0 ± 0.3*	37.2 ± 0.2	36.9 ± 0.2*
Resting HR (beats·min^−1^)	57 ± 8	53 ± 9*	53 ± 8	50 ± 7*
Resting plasma volume (%)	62 ± 2^#^	62 ± 4^#^	57 ± 2	56 ± 5
Haemoglobin (g·dL^−1^)	12.9 ± 0.7^#^	12.9 ± 0.6^#^	14.5 ± 0.7	14.7 ± 0.7
*Running heat tolerance test*				
Peak T_rec_ (°C)	38.8 ± 0.4^#^	38.4 ± 0.3*^#^	38.3 ± 0.3	38.1 ± 0.4*
T_recRISE_ (°C)	1.6 ± 0.3^#^	1.4 ± 0.3*	1.0 ± 0.3	1.1 ± 0.3
Peak T_sk_ (°C)	37.1 ± 0.8^#^	36.1 ± 0.8*^#^	36.0 ± 1.2	35.3 ± 0.7*
Peak HR (beats·min^−1^)	173 ± 22^#^	163 ± 18*^#^	152 ± 13	144 ± 15*
Sweat Loss (%BM)	1.1 ± 0.3^#^	1.3 ± 0.3	1.5 ± 0.7	1.3 ± 0.4
Forearm active sweat glands (per cm^2^)	46 ± 25	66 ± 25*	62 ± 19	58 ± 16
Upper back active sweat glands (per cm^2^)	61 ± 15	64 ± 20	72 ± 18	58 ± 16*
FBF (mL·dL tissue^−1^·min^−1^)	2.77 ± 1.61	3.11 ± 1.80^#^	3.83 ± 2.32	8.30 ± 4.63*

*HR* heart rate, *T*_*rec*_ rectal temperature, *T*_*recRISE*_ rise in rectal temperature, *T*_*sk*_ skin temperature, *BM* body mass, *FBF* forearm blood flow

*Significantly different from Pre (*p* < 0.05); ^#^Significantly different from males at corresponding time-point (*p* < 0.05)

### Running heat tolerance test

#### Body temperatures

**Fig. 2:**
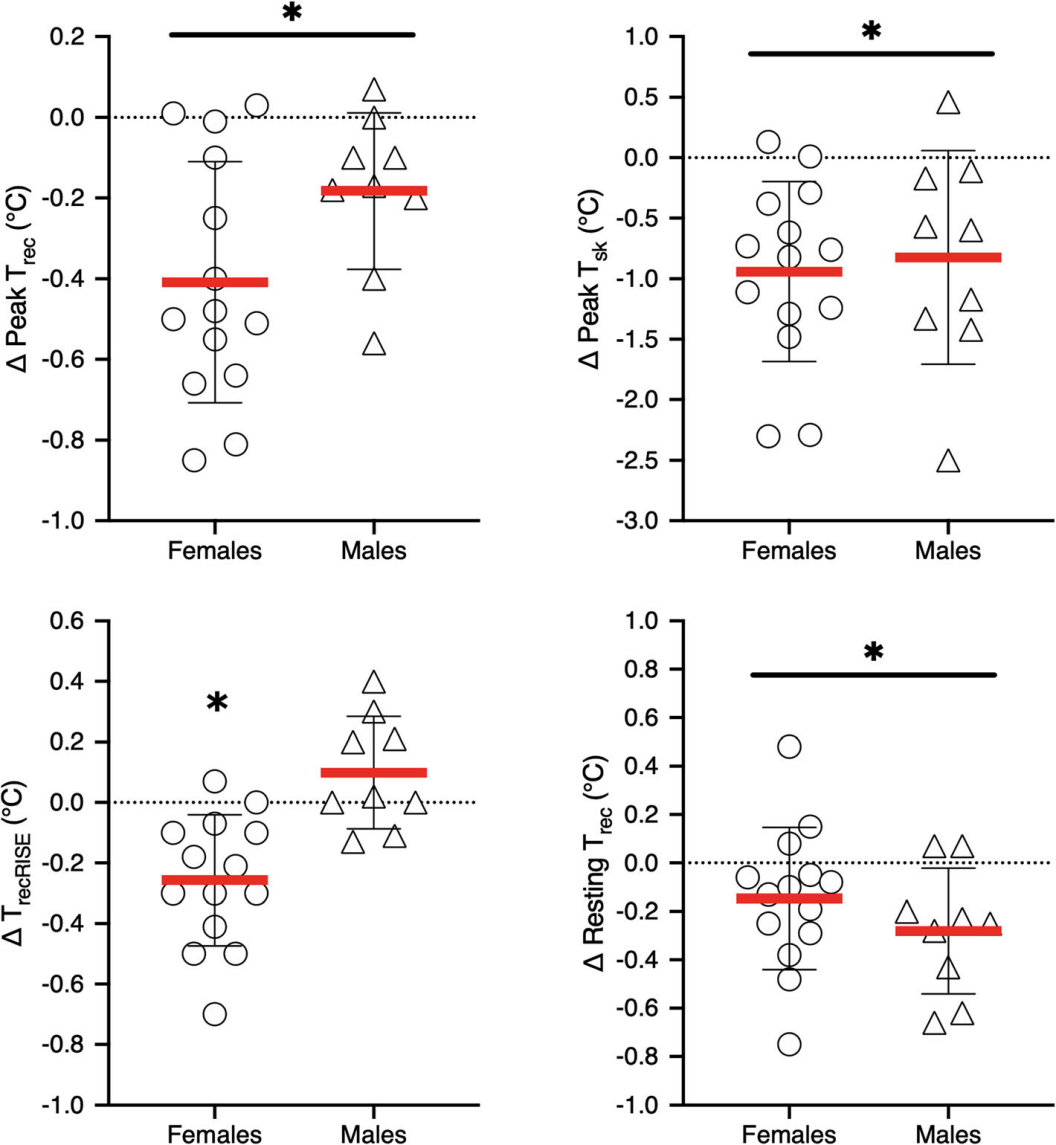
Changes (Δ) in body temperature responses during the running heat tolerance test (HTT; 40 °C, 40%RH) from baseline (Pre) to following (Post) the 3-week post-exercise sauna bathing intervention. Females are represented by circles and males by triangles. Horizontal black lines and error bars represent the group mean ± SD. *Significant difference in Pre vs Post (*p* < 0.05). T_rec_, rectal temperature; T_sk_, skin temperature; T_recRISE_, rise in rectal temperature

Body temperature absolute data and individual participant changes for the SAUNA group are presented in Table 3 and Fig. 2, respectively. Females had a higher peak T_rec_ (0.5 °C [0.2, 0.7], *sex*: *p* < 0.01) and peak T_sk_ (1.1 °C [0.5, 1.7], *sex*: *p* < 0.01) than males across all HTTs. Both females and males reduced peak T_rec_ (− 0.3 °C [− 0.2, − 0.4], *time*: *p* < 0.001) and peak T_sk_ (− 0.9 °C [− 0.5, − 1.2], *time*: *p* < 0.001) from Pre to Post, with a trend for females to experience a greater reduction in peak T_rec_ from Pre to Post (*time × sex*: *p* = 0.06). Peak T_sk_ was reduced to a similar extent in both sexes (*time × sex: p* = 0.66).

There was a *time × sex* interaction on T_recRISE_ (*p* = 0.001) and post hoc comparisons showed that only females reduced T_recRISE_ from Pre to Post (− 0.3 °C [− 0.1, − 0.4], *p*<0.001), whilst T_recRISE_ did not change in the males (+ 0.1 °C [− 0.1, 0.2], *p* = 0.17). Therefore, T_recRISE_ was higher in females than the males at Pre (0.6 °C [0.3, 0.8], *p*<0.001), but not Post (0.2 °C [− 0.1, 0.5], *p* = 0.10).

#### Heart rate

Heart rate absolute data for the SAUNA group are presented in Table 3. Females had a higher peak HR across all HTTs (20 beats·min^−1^ [4, 35], *sex*: *p* = 0.01). However, both females and males experienced a similar reduction in peak HR from Pre to Post (− 9 beats·min^−1^ [− 5, − 14], *time*: *p* = 0.001; *time × sex*: *p* = 0.63).

#### Sweating

**Fig. 3:**
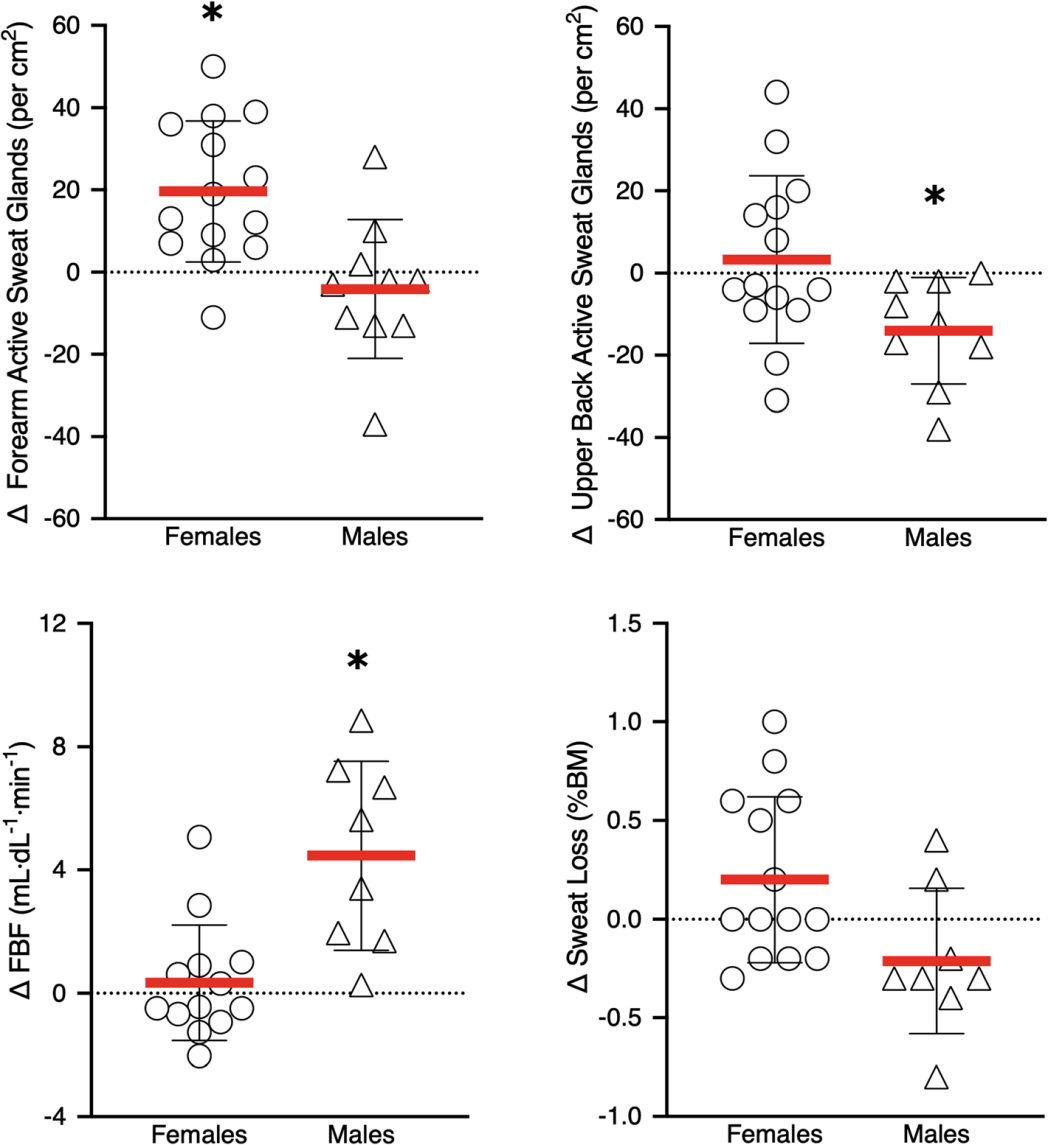
Changes (Δ) in thermoeffector responses during the running heat tolerance test (HTT; 40 °C, 40%RH) from baseline (Pre) to following (Post) the 3-week post-exercise sauna bathing intervention. Females are represented by circles and males by triangles. Horizontal black lines and error bars represent the group mean ± SD *Significant difference in Pre vs Post (*p* < 0.05). FBF, forearm blood flow

Absolute data and individual participant changes in sweating-related outcomes are presented in Table 3 and Fig. 3, respectively. There was a *time × sex* interaction (*p* = 0.03) on sweat loss in the SAUNA group. Post hoc comparisons showed sweat loss was greater in males than in females at Pre (0.5%BM [0.0, 0.9], *p* = 0.05), but not Post (0.0%BM [−0.3, 0.3], *p* = 0.88) sauna intervention. Sweat loss did not change in either females or males from Pre to Post (F: + 0.2%BM [0.0, 0.4], *p* = 0.07; M: −0.2%BM [−0.5, 0.1], *p* = 0.14).

There were significant *time × sex* interactions on forearm (*p* < 0.01) and upper back (*p* = 0.03) sweat gland activity in the SAUNA group. *Post hoc* comparisons showed that females increased sweat gland activity on the forearm by 57% from Pre to Post (+ 20 active sweat glands per cm^2^ [11, 29], *p* < 0.001), but not on the upper back (+3 active sweat glands per cm^2^ [− 7, 13], *p* = 0.51). In contrast, males did not exhibit a change in sweat gland activity on the forearm from Pre to Post (− 4 active sweat glands per cm^2^ [6, −14], *p* = 0.47), and instead decreased sweat gland activity on the upper back by 18% (−14 active sweat glands per cm^2^ [−2, −27], *p* = 0.03). There were no differences between sexes in the SAUNA group in sweat gland activity at Pre or Post, at either site (*p* = 0.12–0.42).

#### Forearm blood flow

FBF showed a *time × sex* interaction (*p* = 0.001) in the SAUNA group (Table 3). Post hoc comparisons showed that males increased FBF by 123% from Pre to Post (+ 4.47 mL·dL tissue^−1^·min^−1^ [2.70, 6.23], *p*<0.001; Fig. 3), whilst FBF remained similar in females (+ 0.34 mL·dL tissue^−1^·min^−1^ [−1.04, 1.73], *p* = 0.61). Consequently, FBF was similar between sexes at Pre (1.07 mL·dL tissue^−1^·min^−1^ [−0.72, 2.86], *p* = 0.23), but was higher in males than in females at Post (5.19 mL·dL tissue^−1^·min^−1^ [2.22, 8.15], *p*<0.01).

#### Perceptual

Overall, females reported higher thermal sensation (*sex*: *p*<0.01) and thermal comfort (*sex*: *p* = 0.001) ratings than males in the SAUNA group (i.e. females reported being hotter and less comfortable). Females and males reported similar reductions in peak thermal sensation (F: 11 ± 1 [“very hot”] and 10 ± 1 [“hot”]; M: 10 ± 1 [“hot”] and 9 ± 1 [“warm”], at Pre and Post, respectively; *time*: *p* < 0.001; *time × sex*: *p* = 0.81) and peak thermal comfort (F: 6 ± 2 [between “uncomfortable” and “very uncomfortable”] and 5 ± 2 [“uncomfortable”]; M: 4 ± 1 [between “slightly uncomfortable” and “uncomfortable”] and 3 ± 1 [“slightly uncomfortable”], at Pre and Post, respectively; *time*: *p* = 0.001; *time × sex*: *p* = 0.08).

Peak RPE_6–20_ showed a *time × sex* interaction (*p* = 0.01) in the SAUNA group. Post hoc comparisons showed that females reduced RPE_6–20_ from Pre to Post (13 ± 3 [“somewhat hard”] to 11±2 [“fairly light”], respectively; *p* = 0.001). In contrast, males reported a similar RPE_6–20_ at Pre and Post (both 10 ± 2 [between “fairly light” and “very light”]; *p* = 0.76). Consequently, females reported higher RPE than the males at Pre (*p* < 0.01), but not Post (*p* = 0.10).

### Temperate exercise tests

#### Lactate profile

In the SAUNA group, both sexes showed a similar increase in running speed at 4 mmol·L^−1^ [La^−^] from Pre to Post (+ 0.37 km·h^−1^ [0.17, 0.57], *time*: *p* = 0.001; *time × sex*: *p* = 0.58; Table 4), though males reached 4 mmol·L^−1^ [La^−^] at a faster running speed than females (3.59 km·h^−1^ [2.58, 4.59], *sex*: *p*<0.001).

**Table 4:** Physiological responses to temperate exercise tests. Data are presented as mean ± SD

	Females		Males	
	*Pre*	*Post*	*Pre*	*Post*
V̇O_2max_ (L·min^−1^)	2.95 ± 0.48^#^	3.10 ± 0.33*^#^	4.34 ± 0.53	4.46 ± 0.49*
V̇O_2max_ (mL·kg^−1^·min^−1^)	52.6 ± 6.9^#^	55.3 ± 4.3*^#^	64.6 ± 2.4	66.6 ± 3.7*
TTE (s)	412 ± 69	450 ± 68*	428 ± 52	448 ± 73*
Speed at 4 mmol·L^−1^ [La^−^] (km·hr^−1^)	14.7 ± 1.5^#^	15.0 ± 1.4*^#^	18.2 ± 0.8	18.6 ± 0.5*
Submaximal HR (beats·min^−1^)	187 ± 11	184 ± 8*	187 ± 11	186 ± 13*
Submaximal RER	1.02 ± 0.06	1.03 ± 0.06	1.05 ± 0.03	1.03 ± 0.04
Submaximal RE (mL·kg^−1^·km^−1^)	190 ± 15	192 ± 13	186 ± 8	189 ± 7

*V̇O*_*2max*_ maximal aerobic capacity, *TTE* time to exhaustion, *[La*^*−*^*]* blood lactate concentration, *HR* heart rate, *RER* respiratory exchange ratio, *RE* running economy

*Significantly different from Pre (*p*<0.05); ^#^Significantly different from males (*p* < 0.05)

#### Submaximal gas exchange and heart rate

Submaximal temperate exercise data for the SAUNA group are presented in Table 4. Submaximal HR (0 beats·min^−1^ [− 9, 7], *sex: p* = 0.87), running economy (1 mL·kg^−1^·km^−1^ [− 5, 12], *sex: p* = 0.37) and RER (+ 0.01 [− 0.03, 0.04], *sex: p* = 0.59) at running speed at 4 mmol·L^−1^ [La^−^] were similar between sexes. Females and males showed a similar reduction in submaximal HR from Pre to Post (− 3 beats·min^−1^ [0, − 6], *time*: *p* = 0.02; *time × sex*: *p* = 0.55). Of note, the ANCOVA comparison between the SAUNA the CON group (Supplementary Material) showed that the SAUNA group only exhibited a trend (*p* = 0.08) for greater changes from Pre to Post in submaximal HR. Submaximal running economy (+3 mL·kg^−1^·km^−1^ [− 4, 9], *time*: *p* = 0.42; *time × sex*: *p* = 0.98) and RER (-0.01 [− 0.02, 0.03], *time*: *p* = 0.59; *time × sex*: *p* = 0.17) did not change from Pre to Post.

#### Heart rate recovery

**Fig. 4:**
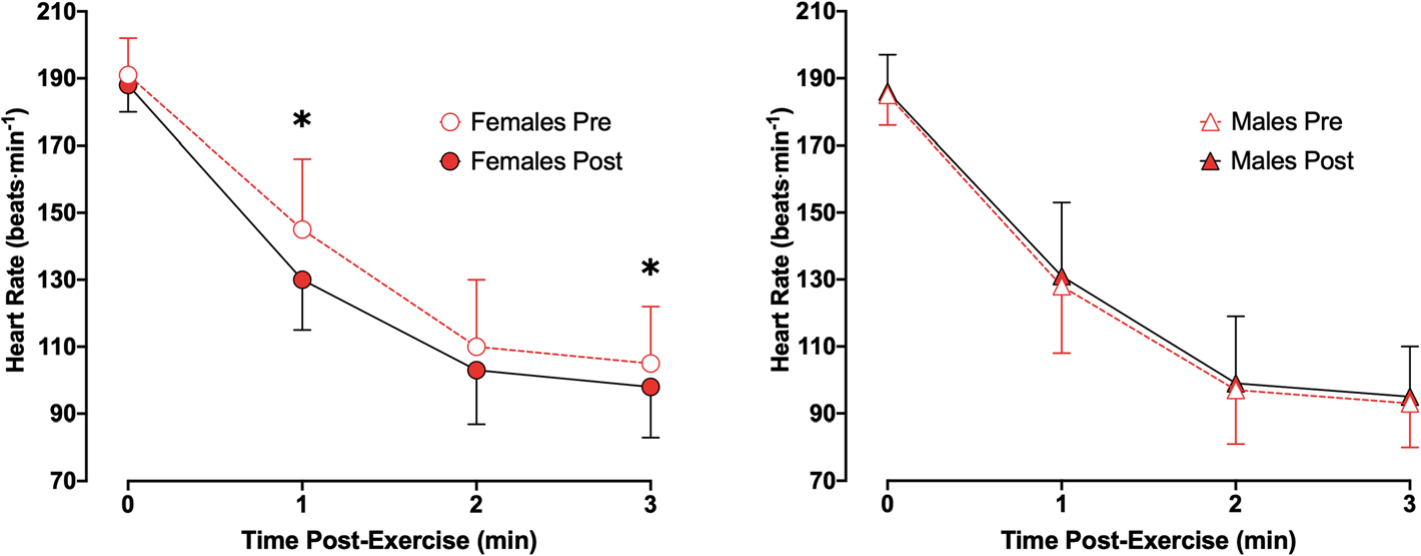
Heart rate recovery from submaximal exercise in temperate conditions (18 °C) in females (*circles*) and males (*triangles*) at baseline (Pre; *open*) vs following (Post; *filled*) the 3-week post-exercise sauna bathing. *Significant difference at Pre vs Post (*p* < 0.05);

There was a three-way interaction of *sex × time × interval* (*p* = 0.04) on HR Recovery in the SAUNA group (Fig. 4). Post hoc comparisons showed that females experienced a reduction in HR at 1 min (*p* < 0.01) and 3 min (*p* = 0.01) from the cessation of exercise, but not significantly so at the immediate cessation (*p* = 0.06) or at 2 min from the cessation of exercise (*p* = 0.07) from Pre to Post. There were no differences at any time-point in males from Pre to Post (*p* = 0.61–0.75).

#### Maximal aerobic capacity test

Females and males showed a similar increase in both absolute (+ 1.3 L·min^−1^ [0.01, 0.25], *time: p* = 0.03; *time × sex*: *p* = 0.86) and relative (+ 2.4 mL·kg^−1^·min^−1^ [0.4, 4.3], *time: p* = 0.02; *time × sex*: *p* = 0.73) V̇O_2max_ from Pre to Post (Table 4). However, both absolute and relative V̇O_2max_ was higher in males than in females (by 1.38 L·min^−1^ [1.02, 1.73], and 11.6 mL·kg^−1^·min^−1^ [8.0, 15.3], respectively; *sex*: both *p* < 0.001). In addition, females and males showed similar increases (~ 8%) in TTE in the V̇O_2max_ test (+ 29 s [14, 43], *time*: *p* < 0.001; *time × sex*: *p* = 0.20) from Pre to Post (Table 4). TTE was not different between sexes (7 s [− 47, 61], *sex*: *p* = 0.79).

## Discussion

The main findings of this study were that, contrary to the first hypothesis, both female and male endurance athletes exhibited similar physiological strain during an acute bout of post-exercise sauna bathing. Furthermore, contrary to the second hypothesis, 3 weeks of intermittent post-exercise sauna bathing was *not* more effective in males. In fact, post-exercise sauna bathing was possibly more effective in female athletes, as both sexes showed similar reductions in thermal and cardiovascular strain whilst only females showed an attenuated *rise* in rectal temperature during exercise-heat stress at post-intervention. Moreover, there were sex differences in thermoeffector adaptations. Males exhibited marked increases in forearm blood flow in response to exercise-heat stress, as well as reduced sweat gland activity on the back but not the forearm post-intervention. Conversely, females exhibited increased sweat gland activity on the forearm and no changes on the back and did not experience any changes in forearm blood flow.

Temperate performance marker results also contradicted the second hypothesis, as both sexes in the SAUNA group showed similar improvements in markers of temperate exercise performance (i.e. V̇O_2max_, running speed at 4 mmol·L^−1^ [La^−^], TTE and submaximal HR) following 3 weeks of intermittent post-exercise sauna bathing. HR recovery from submaximal temperate exercise was faster at post- as compared to pre-intervention in SAUNA females only. Changes in temperate performance markers in the SAUNA group were greater than in the CON group undergoing 3 weeks of normal training, which indicates that improvements in both sexes in the SAUNA group were as a result of the post-exercise sauna intervention.

### Intervention and training

Overall, the post-exercise sauna bathing intervention appeared to invoke a similar physiological stimulus in the subset of females and males sampled at the first sauna session (i.e. similar T_rec_, HR, sweat loss and thirst, as indexed by similar ad libitum fluid consumption), as well as in those observed during the course of the intervention (i.e. similar HR). These similarities were somewhat unexpected, considering females likely experienced greater dry heat gain in the hot–dry conditions of the sauna because of their greater surface-area-to-mass ratio [13]. Furthermore, both sexes entered the sauna at T_rec_ ~ 37.6 °C, despite female participants running at a higher mean HR than male participants in the training session immediately before the first post-exercise sauna bathing session. Peak T_rec_ in the sauna was also similar between sexes at the first post-exercise sauna session, reaching ~ 38.5 °C, the suggested core body temperature threshold for inducing thermoregulatory adaptations [38].

It should be noted that the first post-exercise sauna session, and most of those thereafter, occurred in the evenings (19:00–20:00), when circadian rhythms would have aided in reaching the ~ 38.5 °C T_rec_ threshold [6, 39]. Still, T_rec_ observed at the first sauna session may have underestimated T_rec_ at subsequent sessions. Instrumentation and baseline measurements at the first sauna session caused a considerable delay (~ 17 min) between the end of exercise and entering the sauna. Participants entered the sauna almost immediately post-exercise in subsequent sessions, and thus likely entered (and exited) the sauna at a higher T_rec_ than that observed in the first session. Furthermore, rectal temperature is known to be slower to respond to rapid increases in core temperature as compared to other methods (e.g. oesophageal or telemetric pill [40]). Consequently, participants’ “true” core temperature was likely higher than that measured when they exited the sauna.

Whilst participants reached a peak T_rec_ of at least ~38.5 °C in the first sauna session, it is likely that the thermal impulse (i.e. elevated T_rec_) diminished as the protocol progressed [41]. Indeed, Tyka et al. [42] observed that males’ peak T_rec_ during a sauna bath (independent of exercise) was ~ 38.5 °C at the first session, but was reduced to, and subsequently plateaued, at ~ 37.8 °C by the fifth session. It is also possible that T_rec_ was lower upon entering the sauna after exercise by the end of the protocol, since Zurawlew et al. [20] showed that 6 days of post-exercise hot water immersion reduced peak T_rec_ during exercise in 18 °C. Nonetheless, heat acclimation adaptations were observed despite thermal impulse likely waning by the latter sauna exposures.

Lastly, despite different overall running distances in training, there were no differences in weekly type, frequency and perceived exertion of sessions between sexes. Session RPE, especially when combined with other training parameters, has been suggested as a reliable method for training load monitoring [43]. Thus, these data indicate a similar relative training stimulus, especially as participants were from the same running club.

### Exercise-heat tolerance

Both females and males in the SAUNA group showed similar reductions in peak and resting T_rec_ post-intervention, with mean reductions reaching or exceeding the 0.2 °C threshold identified as physiologically meaningful [44]. Both sexes in the SAUNA group also reduced peak T_sk_ and peak HR to a similar extent, with the rate of rise in T_rec_ (i.e. T_recRISE_) attenuated post-intervention in the females only. That females showed thermoregulatory and cardiovascular adaptations similar, or sometimes superior to males, was contrary to the hypothesis, which was informed by the robust sex-comparison study by Mee et al. [16]. To achieve these adaptations, female and male participants in the current study received ~ 275 min of exogenous, passive heat exposure via post-exercise sauna bathing in addition to their normal training over a 3-week period. In contrast, Mee et al. [16] used a traditional isothermic heat acclimation protocol (T_rec_ rapidly elevated and maintained at ≥ 38.5 °C) and found that 5 consecutive days of 90-min active heat exposures (i.e. a total 450 min of heat exposure) resulted in body temperature and cardiovascular adaptations in males but not in females. Mee and colleagues showed that females required 10 days (and thus 900 min) of isothermic heat exposure to achieve the same level of adaption as their male counterparts (and a similar degree of adaptation as demonstrated by females in the current study). By design, the current study used the same heat tolerance test as Mee et al. [16] to examine heat adaptation to allow us to compare the magnitude of adaptation. We observed a ~ 0.4 °C reduction in peak T_rec_, ~ 1.0 °C reduction in T_sk_ and ~10 beats·min^−1^ reduction in HR in our female cohort, similar to the ~ 0.5 °C, ~ 1.1°C and ~10 beats·min^−1^ reductions, respectively, reported in females by Mee et al. [16] following 10 days of isothermic heat acclimation. These similarities were in spite of ~ 70% less total exogenous heat exposure time in the current study as compared to the 10-consecutive day heat acclimation protocol by Mee et al. [16], albeit the 10 exposures in the current study were spread across 3 weeks. Whether sex differences similar to that observed by Mee et al. [16] would have been apparent after 5 post-exercise sauna bathing sessions (i.e. ~ 1.5 weeks) is unknown. However, the higher fitness level of female participants in the current study as compared to that by Mee et al. [16] (~ 53 vs ~ 45 mL·kg^−1^·min^−1^) may have facilitated a faster rate of adaptation [45].

It appears that the reduction in peak T_rec_ in males was achieved through a lower resting T_rec_ alone. Yet, females achieved a reduction in peak T_rec_ through both a lowered resting T_rec_ and a reduced T_recRISE_ during the HTT, culminating in the trend for a greater reduction in peak T_rec_ in females. Nonetheless, the attenuated T_recRISE_ in the SAUNA group females and the lack of change in T_recRISE_ in males may have been influenced by the tendency for participants with “worse” baseline values (i.e. females) to show a greater improvement. The fixed workload of the HTT was not specific to sex, fitness or anthropometry. Males were therefore working at a lower intensity relative to their fitness level and at a lower metabolic heat production relative to their larger body size than females [46]. Consequently, males in the SAUNA group consistently achieved lower T_recRISE_ values at baseline than females and would need to show a greater relative improvement in heat dissipation and/or reduction in heat production to exhibit the same absolute improvement in T_recRISE_ as females. These differences in metabolic heat production as well as relative intensity make it somewhat difficult to compare the magnitude of the change in core body temperatures between sexes.

Only females in the SAUNA group increased sweat gland activity on the forearm post-intervention. Data from our laboratory [47] also showed an increase in forearm sweat gland activity in a female cohort following consecutive-day heat acclimation. However, in the current study, which is consistent with others [48–50], males showed no change in forearm sweat gland activation following repeated heat exposures. Thus, the increase in forearm sweat gland activity in our previous post-exercise sauna investigation [7] was likely driven by the predominantly female cohort (9F, 3M). Considering females exhibit relatively lower sudomotor thermosensitivity and maximal sweat output per gland [11], the increase in sweat gland activity observed in the current study may be due to peripheral changes to the cholinergic sensitivity of the forearm sweat glands or the size of the sweat glands in response to heat acclimation, allowing more active sweat glands to be identified [51, 52]. Though mechanisms are yet to be delineated, adaptations that increase the number of active sweat glands on the upper limbs appear to be unique to female cohorts undergoing heat acclimation. Curiously, this 57% increase in forearm sweat gland activation in females was observed without an increase in total sweat loss. This may indicate a more efficient distribution of sweating, whereby the relative contribution to whole-body sweat loss is shifted away from the back and towards the upper limbs following acclimation, as observed by Smith and Havenith [53]. Moreover, it is possible that there was no change in whole-body sweat loss in the current study because the post-exercise sauna bathing intervention probably did not maintain thermal impulse throughout (i.e. T_rec_ ~ 38.5 °C [38]), as discussed above. Maintaining thermal impulse by using an isothermic heat acclimation protocol has previously been suggested to induce preferential sudomotor adaptations [54, 55], regardless of whether heat acclimation is induced using an active or passive model [56].

Only males in the SAUNA group increased FBF post-intervention, which is consistent with what others have shown in male cohorts following active heat acclimation [57–59]. This increase in FBF was likely a result of improved cutaneous vascular function [60], which is largely nitric oxide-dependent [61]. Females are less reliant on nitric oxide-dependent dilation to increase blood flow to the skin during local heating, as oestrogen can stimulate vasodilation in a nitric oxide-independent manner [62, 63], which might limit these functional adaptations. Of note, whilst females and males are known to exhibit similar skin blood flow responses to passive heating at the forearm, females show markedly greater skin blood flow at the thigh [64]. Thus, the single-site measurement in the current study may not be representative of whole-body changes in skin blood flow. Nevertheless, it should be stipulated that although females did not increase FBF following the post-exercise sauna intervention, they achieved a similar FBF at a ~ 0.4 °C lower T_rec_, indicating greater vasomotor thermosensitivity.

As previously noted, the fixed external workload (9 km·h^−1^, 2% grade) of the HTT likely resulted in different rates of metabolic heat production between the female and male cohorts, given they were significantly different physical sizes (i.e. body mass and body surface area [46]). This makes it somewhat difficult to attribute sex differences in thermoeffector outcomes to the intervention alone. It is possible that the different heat loads experienced during the HTT confounded observations of forearm blood flow and sweat gland activity. Whilst there were distinctive sex differences in these thermoeffector responses following the SAUNA intervention in the current study, whether this truly represents an adaptive sex difference should be further investigated using a heat tolerance test matching metabolic heat production and heat load. Lastly, the physiological and perceptual adaptations observed during the HTT imply that exercise performance (e.g. a time trial) in the heat would be improved. However, this fixed-workload model does not give insight as to how these expected improvements would compare to those gained via an active heat acclimation intervention. A passive heating protocol also does not replicate the perceptual experience of exercising in the heat and does not allow an athlete to trial race-day strategies surrounding pacing, hydration and fuelling.

### Temperate exercise performance

Both females and males in the SAUNA group improved absolute and relative V̇O_2max_, running speed at 4 mmol·L^−1^ [La^−^] and TTE to a similar extent. Previously, we showed in a subset of the participants (mixed-sex cohort; included in the current study) that 3 weeks of intermittent post-exercise sauna bathing was effective for improving markers of submaximal and maximal temperate exercise performance [7]. With the addition of more participants of both sexes, this study further supports the literature evidencing the ergogenic effects of heat acclimation, and importantly, it supports the ergogenic benefits of heat acclimation in both sexes.

Quantification (and debate) of improvements in exercise performance and V̇O_2max_ in relatively cooler conditions following heat acclimation have been mainly limited to male data. Only two studies to date have investigated changes in V̇O_2max_ following heat acclimation in a female cohort [65, 66]. Fortney and Senay [65] employed 90-min cycling at 30%V̇O_2max_ in 45 °C, 30%RH daily for 2 weeks, then every other day for an additional 2 weeks, and did not observe any changes in V̇O_2max_. Conversely, Bailey et al. [66] employed 30-min hot-water immersion (42 °C water to the chest) thrice weekly for 8 weeks and observed a 5.6% improvement in V̇O_2max_. This is similar to the ~ 5% improvement in females in the current study. However, participants in both previous studies were sedentary and/or untrained. Thus, the current study provides the first evidence of improved V̇O_2max_ following heat acclimation in a trained female cohort. Moreover, the current study shows that this improvement is similar to that experienced in a male cohort training within the same running club. Thus, the female cohort in the current study did not show a reduced trainability of V̇O_2max_, which is contrary to the exercise-intervention data in females that informed the study’s hypothesis [22].

Females and males in the current study, given a similar relative training stimulus and a similar thermal stimulus in the sauna, showed similar improvements in speed at 4 mmol·L^−1^ [La^−^]. This is supported by previous research, showing a similar reduction in submaximal exercise metabolism in males and females exercising in cool conditions following heat acclimation [67]. Furthermore, Hafen et al. [68] reported increases in mitochondrial biogenesis and function (the mechanisms expected to mainly underpin improvements in speed at 4 mmol·L^−1^ [La^−^] [69]) following 6 days of 2-h localised thigh heating in a mixed-sex cohort (10 F, 10 M). However, the current study is the first to specifically investigate the effects of sex on metabolic thresholds in temperate conditions following heat acclimation. Neither males nor females in the control group experienced improvements in these performance outcomes, which indicates that 3 weeks of normal training alone was not sufficient to improve V̇O_2max_, running speed at 4 mmol·L^−1^ [La^−^] or TTE in this well-trained cohort.

Only females in the SAUNA group had a faster HR recovery in temperate conditions post-intervention. Pre- to post-intervention differences were especially pronounced at 60 s post-exercise (i.e. the “fast phase” of HR recovery), whereby vagal reactivation is the predominant neural factor controlling HR recovery post-exercise [70]. Increased autonomic, and specifically vagal, control of the heart characterises the initial transient phase of the biphasic pattern of heat acclimation [71, 72]. Indeed, a faster HR recovery 60 s post-submaximal temperate exercise was previously observed in males following a 10-consecutive-day post-exercise sauna bathing protocol [9]. However, in the current study, males in the SAUNA group did not show changes in HR recovery post-intervention, possibly because the sauna exposures were spread over 3 weeks. A pattern of initial increase in autonomic excitability, followed by autonomic withdrawal has previously been shown when using ~ 3 weeks of heat acclimatisation in males [73]. Thus, perhaps in the male cohort in the current study, the 3-week, intermittent nature of the intervention allowed time for more stable cardiovascular adaptations to underpin changes in HR (i.e. enhanced physiological efficiency and decreased excitability, in line with characteristics of the second, stabilisation phase of heat acclimation [72]), whilst females, who have been shown to take longer to develop the initial cardiovascular adaptations in response to heat acclimation [72], remained in the initial transient phase of adaptation at post-intervention. Sex differences in autonomic control of the cardiovascular system in response to various stressors (e.g. exercise, hypoxia, head-up tilt) are well-documented [74] and may affect the biphasic response to heat acclimation.

There were no changes in plasma volume measures in either group. However, it was somewhat unlikely that a heat-mediated shift in relative plasma volume (observed as quickly as after the first two heat exposures [75]) would persist for the duration of the 3-week intervention. As detailed in the investigation by Stanley and colleagues [9], relative plasma volume shifts were no longer significantly different from baseline by the sixth consecutive day of post-exercise sauna bathing, and mean values returned to baseline by the ninth day. Similarly, whilst Scoon et al. [8] observed increases in *absolute* plasma volume following 3 weeks of intermittent post-exercise sauna bathing, haemoglobin concentration and haematocrit (the measures from which our plasma volume data were derived), were the same at pre- and post-intervention. Considering similarities between the current protocol and that of Scoon et al. [8], it is likely that participants experienced absolute increases in plasma volume and that plasma volume expansion underpinned performance improvements to some extent [76]. Notably, Scoon et al. [8] also observed an increase in total haemoglobin mass using 3 weeks of intermittent post-exercise sauna bathing. Though not measured in the current study, it is possible that increases in total haemoglobin mass further aided improvements in V̇O_2max_ in this cohort, especially since iron supplements would have supported any erythropoietic stimuli from the training and sauna intervention.

### Perspectives and considerations

Given current recommendations for athletes to utilise post-exercise sauna bathing to prepare for athletic events in the heat [4, 5] plus the increased interest in using heat as an ergogenic aid [18, 19], these data are important because they confirm that post-exercise sauna bathing is an appropriate and effective training intervention for both sexes *and* in a trained population. The accessible and pragmatic nature of post-exercise sauna bathing makes these data especially meaningful for female athletes, who may not otherwise have access to traditional methods of heat acclimation involving climatic chambers or to other costly ergogenic aids, given the comparatively limited funding at present in some female sporting environments [77]. For application purposes, future studies should also build on these findings by investigating the decay/retention of the adaptations accrued using an intermittent post-exercise sauna bathing intervention.

The random sampling of the female cohort across menstrual cycle phases and hormonal contraceptive use provides greater confidence in the ecological validity of these findings for female athletes because females were not tested in just one menstrual cycle phase [78]. The so-called flipside of the enhanced ecological validity is that there may be greater associated variability. Additionally, this meant we did not have the opportunity to gain insight into the underlying effects (if any) of endogenous or exogenous female sex hormones on our results [79]. However, it was deemed *most* important to structure the protocol around the club’s training schedule to ensure a similar exercise training stimulus between groups and sexes.

It is possible that female participants may have experienced a greater thermal stimulus when undergoing post-exercise sauna bathing during their luteal phase. However, as body temperatures were not measured after the first post-exercise sauna bathing session, we cannot ascertain this. Nevertheless, overall peak HR in the sauna (somewhat indicative of thermal strain) was not different between sexes across the duration of the intervention.

Two female SAUNA group participants completed their HTTs in different menstrual phases. This likely caused very minor, if any, variation in the results of the total 14 females in the SAUNA group who completed both HTTs. With regards to temperate exercise tests, a recent investigation of submaximal and maximal exercise responses in oral contraceptive users and non-users showed no effects of either menstrual cycle phase or oral contraceptive pill phase (i.e. active or inactive) on V̇O_2max_, TTE, [La^−^] nor submaximal gas exchange or HR [80]. Therefore, differences in the menstrual cycle phase from Pre to Post in 3 out of 16 female participants were unlikely to have affected temperate exercise findings.

Finally, due to the nature of the running HTT, FBF was assessed post-exercise. The ~5 min period immediately post-exercise is associated with dynamic changes in heart rate and blood pressure, amongst other haemodynamic variables [81]; although cardiovascular responses during recovery from exercise-heat stress are similar between males and females [82]. Thus, whilst every effort was made to replicate the FBF measurement conditions (i.e. only ~ 26-s difference between Pre and Post FBF measurement timing), it is possible that the adaptive sex differences observed in these data may not necessary reflect FBF changes *during* exercise.

## Conclusions

The findings from the current study demonstrate that, in an ecologically valid setting, females and males demonstrated similar cardiovascular, thermoregulatory, sweat and thirst responses during an acute session of post-exercise sauna bathing. Chronically, 3 weeks of post-exercise sauna bathing resulted in similar HR and skin and core body temperature reductions during exercise-heat stress in both female and male endurance athletes, which indicates a similar level of improvement in these main markers of heat adaptation. Although there appeared to be sex differences in thermoeffector adaptations (i.e. only males increased FBF and only females increased forearm sweat gland activity), and only females showed a reduced rise in T_rec_ during exercise-heat stress, it is unclear how differences in metabolic heat production due to the fixed work-load of the HTT may have contributed to the observed sex differences. However, there were also sex differences in HR recovery from submaximal temperate exercise, in that only females showed a more rapid HR recovery post-intervention. It is possible that this indicates sex differences in the temporal patterning of underlying autonomic heat acclimation adaptions. Lastly, post-exercise sauna bathing induced similar meaningful improvements in temperate exercise performance in female and male athletes, as evidenced by both sexes showing similar changes in V̇O_2max_, running speed at 4 mmol·L^−1^ [La^−^] and TTE following the post-exercise sauna intervention. Taken together, the findings of this study highlight that post-exercise sauna bathing is an effective method of heat acclimation and an ergogenic aid for both sexes.

## Supplementary Information

**Additional file 1.**

## Data Availability

Data available on request.

## Abbreviations

[La^−^]: Blood lactate concentration
ANOVA: Analysis of variance
CON: Control group
FBF: Forearm blood flow
Hb: Haemoglobin
Hct: Haematocrit
HR: Heart rate
HTT: Running heat tolerance test
Pre: Pre-intervention tests
Post: Post-intervention tests
RER: Respiratory exchange ratio
RPE: Rating of perceived exertion
SAUNA: Sauna intervention group
T_rec_: Rectal temperature
T_sk_: Mean weighted skin temperature
TTE: Time-to-exhaustion
V̇O_2max_: Maximal aerobic capacity
