# Supplementary material for: Sex differences in adaptation to intermittent post-exercise sauna bathing in trained middle-distance runners
Source: Sports Med Open. 2021 Jul 23;7:51. doi: 10.1186/s40798-021-00342-6 (PMC8302716; doi:10.1186/s40798-021-00342-6)
Supplement: Supplementary file 1 — Additional file 1. [file 40798_2021_342_MOESM1_ESM.docx]

**Supplementary Materials**

**Sex differences in adaptation to intermittent post-exercise sauna bathing in trained middle-distance runners**

Authors:

*Nathalie V. Kirby^1,2^

Samuel J. E. Lucas^1^

Thomas G. Cable^1,3^

Oliver J. Armstrong^4^

Samuel R. Weaver^1^

Rebekah A. I. Lucas^1^

^1^University of Birmingham, Birmingham, United Kingdom

^2^Human and Environmental Physiology Research Unit, University of Ottawa, Ottawa, Canada

^3^Loughborough University, Loughborough, United Kingdom

^4^Performance Centre, University of Birmingham Sport, Birmingham, United Kingdom

*Corresponding author: Nathalie V. Kirby

[nkirby@uottawa.ca](mailto:nkirby@uottawa.ca)

University of Ottawa, K1N 6N5

There was no effect of *sex* (*p*=0.111-0.686) or *group* (*p*=0.166-0.853) and no interaction effects (*p*=0.234-0.938) on the weekly frequency of each type of training session. There was no effect of *sex* (*p*=0.409-0.926) or *group* (*p*=0.233-0.774) and no interaction effects (*p*=0.156-0.793) on RPE_1-10_ of each type of training session. Weekly running distance was greater overall in males than females (*sex:* *p*<0.001), but there was no main effect of *group* (*p*=0.628) or interaction effect (*p*=0.616).

**Supplementary Table 1** Weekly training type, frequency, mean perceived exertion and running distance during the intervention period. Data are presented as mean±SD

|  |  | SAUNA | | |  | CON | |
| --- | --- | --- | --- | --- | --- | --- | --- |
| *Session Type* |  | *Frequency (weekly)* | *RPE_1-10_* |  | *Frequency (weekly)* | | *RPE_1-10_* |
| Easy | *F* | 2±1 | 4±1 |  | 2±1 | | 4±1 |
|  | *M* | 2±1 | 3±1 |  | 2±1 | | 4±1 |
| Tempo | *F* | 1±0 | 6±1 |  | 1±1 | | 6±1 |
|  | *M* | 1±0 | 6±1 |  | 1±0 | | 7±1 |
| High-Intensity | *F* | 2±1 | 8±1 |  | 2±1 | | 8±1 |
|  | *M* | 2±0 | 8±1 |  | 2±1 | | 8±0 |
| Long | *F* | 1±0 | 5±1 |  | 1±0 | | 4±2 |
|  | *M* | 1±0 | 5±1 |  | 1±0 | | 5±1 |
|  |  | *Weekly Distance (km)* | |  | *Weekly Distance (km)* | | |
| Total* | *F* | 42.8±17.1 | |  | 42.9±12.8 | | |
|  | *M* | 69.5±13.0 | |  | 63.2±27.3 | | |

SAUNA, post-exercise sauna bathing intervention group; CON, control group; RPE_1-10_, mean rating of perceived exertion on a 1-10-point scale. Analysed using a two-way (*group* *× sex*) analysis of variance. *Significant main effect of *sex* (*p*<0.001)

|  | **Supplementary Table 2** Physiological responses at rest and during the Running Heat Tolerance Test. Absolute data are presented as mean±SD. Changes (Δ) represent mean differences. *P*-values represent main effects of sex and intervention group, and sex × group interaction effects | | | | | | | | | | | | | |
| --- | --- | --- | --- | --- | --- | --- | --- | --- | --- | --- | --- | --- | --- | --- |
|  | |  | SAUNA | | |  | CON | | |  | *P*-value | | |  |
| ***Rest*** | |  | *Pre* | *Post* | *Δ* |  | *Pre* | *Post* | *Δ* |  | *Sex* | *Group* | *Interaction* |  |
| Resting T_rec_ (°C) | | *F* | 37.2±0.1 | 37.0±0.3 | -0.2 |  | 37.2±0.4 | 37.4±0.6 | +0.2 |  | 0.260 | **0.007** | 0.950 |  |
|  |  | *M* | 37.2±0.2 | 36.9±0.2 | -0.3 |  | 37.3±0.2 | 37.3±0.3 | 0.0 |  |  |  |  |  |
| Resting HR (beats⋅min^-1^) | | *F* | 57±8 | 53±9 | -4 |  | 54±6 | 51±6 | -3 |  | 0.659 | 0.632 | 0.765 |  |
|  |  | *M* | 53±8 | 50±7 | -3 |  | 53±6 | 51±5 | -3 |  |  |  |  |  |
| Resting Plasma Volume (%) | | *F* | 62±2 | 62±4 | 0 |  | 61±2 | 61±3 | 0 |  | 0.095 | 0.429 | 0.086 |  |
|  |  | *M* | 57±2 | 56±5 | -1 |  | 58±3 | 61±7 | +3 |  |  |  |  |  |
| ***Running Heat Tolerance Test*** | | |  |  |  |  |  |  |  |  |  |  |  |  |
| Peak T_rec_ (°C) | | *F* | 38.8±0.4 | 38.4±0.3 | -0.4 |  | 38.5±0.5 | 38.7±0.5 | +0.2 |  | 0.130 | **0.011** | 0.325 |  |
|  |  | *M* | 38.3±0.3 | 38.1±0.4 | -0.2 |  | 38.6±0.3 | 38.4±0.3 | -0.2 |  |  |  |  |  |
| T_recRISE_ (°C) | | *F* | 1.6±0.3 | 1.4±0.3 | -0.2* |  | 1.4±0.5 | 1.4±0.4 | 0.0 |  | 0.170 | 0.834 | **0.046** |  |
|  |  | *M* | 1.0±0.3 | 1.1±0.3 | +0.1 |  | 1.3±0.4 | 1.2±0.5 | -0.1 |  |  |  |  |  |
| Peak T_sk_ (°C) | | *F* | 37.1±0.8 | 36.1±0.8 | -1.0 |  | 36.7±0.9 | 36.8±0.8 | +0.1 |  | 0.061 | **<0.001** | 0.615 |  |
|  |  | *M* | 36.0±1.2 | 35.3±0.7 | -0.7 |  | 36.2±1.3 | 36.0±1.3 | -0.2 |  |  |  |  |  |
| Peak HR (beats⋅min^-1^) | | *F* | 173±22 | 163±18 | -10 |  | 168±11 | 166±18 | -2 |  | 0.394 | **0.039** | 0.950 |  |
|  |  | *M* | 152±13 | 144±15 | -8 |  | 153±12 | 152±10 | -1 |  |  |  |  |  |
| Sweat Loss (%BM) | | *F* | 1.1±0.3 | 1.3±0.3 | +0.2 |  | 1.3±0.4 | 1.0±0.3 | -0.3 |  | 0.544 | **0.035** | 0.080 |  |
|  |  | *M* | 1.5±0.7 | 1.3±0.4 | -0.2 |  | 1.4±0.5 | 1.2±0.2 | -0.2 |  |  |  |  |  |
| Forearm active sweat glands (per cm^2^) | | *F* | 46±25 | 66±25 | +20 |  | 44±13 | 40±20 | -4 |  | 0.061 | **0.004** | 0.275 |  |
|  |  | *M* | 62±19 | 58±16 | -4 |  | 48±20 | 37±20 | -11 |  |  |  |  |  |
| Upper back active sweat glands (per cm^2^) | | *F* | 61±15 | 64±20 | +3 |  | 78±14 | 64±29 | -14 |  | 0.085 | 0.187 | 0.983 |  |
|  |  | *M* | 72±18 | 58±16 | -14 |  | 61±13 | 42±4 | -19 |  |  |  |  |  |
| FBF (mL⋅dL tissue^-1^⋅min^-1^) | | *F* | 2.77±1.61 | 3.11±1.80 | +0.34 |  | 2.59±1.89 | 2.49±0.92 | -0.10 |  | **0.029** | **0.018** | 0.058 |  |
|  |  | *M* | 3.83±2.32 | 8.30±4.63 | +4.47 |  | 5.54±1.97 | 4.81±2.49 | -0.73 |  |  |  |  |  |

SAUNA, post-exercise sauna bathing intervention group; CON, control group; F, females; M, males; HR, heart rate; T_rec_, rectal temperature; T_recRISE_, rise in rectal temperature; T_sk_, skin temperature; BM, body mass; FBF, forearm blood flow. Analysed using two-way (*group* *× sex*) analysis of covariance of change scores with baseline scores as a covariate. *Post hoc* analyses: *significantly different from Δ in SAUNA males (*p*<0.05)

| **Supplementary Table 3** Physiological responses to temperate exercise tests. Absolute data are presented as mean±SD. Changes (Δ) represent mean differences. *P*-values represent main effects of sex and intervention group, and sex × group interaction effects | | | | | | | | | | | | | | |
| --- | --- | --- | --- | --- | --- | --- | --- | --- | --- | --- | --- | --- | --- | --- |
|  |  | SAUNA | | | |  | CON | | |  | *P*-value | | | |
|  |  | | *Pre* | *Post* | *Δ* |  | *Pre* | *Post* | *Δ* |  | *Sex* | *Group* | *Interaction* |  |
| V̇O_2max_ (L⋅min^-1^) | *F* | | 2.95±0.48 | 3.10±0.33 | +0.14 |  | 2.83±0.20 | 2.86±0.35 | +0.03 |  | **0.005** | **0.016** | 0.572 |  |
|  | *M* | 4.34±0.53 | | 4.46±0.49 | +0.12 |  | 4.06±0.37 | 4.03±0.19 | -0.03 |  |  |  |  |  |
| V̇O_2max_ (mL⋅kg^-1^⋅min^-1^) | *F* | 52.6±6.9 | | 55.3±4.3 | +2.7 |  | 52.2±4.1 | 52.6±2.3 | +0.4 |  | **0.001** | **0.031** | 0.996 |  |
|  | *M* | 64.6±2.4 | | 66.6±3.7 | +2.0 |  | 63.6±3.2 | 63.6±4.5 | 0.0 |  |  |  |  |  |
| TTE (s) | *F* | 412±69 | | 450±68 | +38 |  | 419±72 | 416±76 | -3 |  | 0.246 | **0.006** | 0.890 |  |
|  | *M* | 428±52 | | 448±73 | +20 |  | 409±64 | 400±72 | -9 |  |  |  |  |  |
| Speed at 4 mmol·L^-1^ [La^-^] (km⋅hr^-1^) | *F* | 14.7±1.5 | | 15.0±1.4 | +0.3 |  | 15.1±0.9 | 15.2±0.6 | +0.1 |  | **0.043** | **0.029** | 0.219 |  |
|  | *M* | 18.2±0.8 | | 18.6±0.5 | +0.4 |  | 17.7±1.0 | 17.7±1.0 | 0.0 |  |  |  |  |  |
| Submaximal HR (beats⋅min^-1^) | *F* | 187±11 | | 184±8 | -3 |  | 187±4 | 188±8 | +1 |  | 0.479 | 0.079 | 0.774 |  |
|  | *M* | 187±11 | | 186±13 | -1 |  | 178±12 | 180±11 | +2 |  |  |  |  |  |
| Submaximal RER | *F* | 1.02±0.06 | | 1.03±0.06 | +0.01 |  | 1.02±0.04 | 1.04±0.04 | +0.02 |  | 0.813 | 0.488 | 0.210 |  |
|  | *M* | 1.05±0.03 | | 1.03±0.04 | -0.02 |  | 1.04±0.03 | 1.05±0.04 | +0.01 |  |  |  |  |  |
| Submaximal RE  (mL⋅kg^-1^⋅km^-1^) | *F* | 190±15 | | 192±13 | +2 |  | 178±22 | 187±18 | +9 |  | 0.996 | 0.844 | 0.600 |  |
|  | *M* | 186±8 | | 189±7 | +3 |  | 186±11 | 191±10 | +5 |  |  |  |  |  |

SAUNA, post-exercise sauna bathing intervention group; CON, control group; F, females; M, males; V̇O_2max_, maximal aerobic capacity; TTE, time to exhaustion; [La^-^], blood lactate concentration; HR, heart rate; RER, respiratory exchange ratio; RE, running economy. Analysed using two-way (*group* *× sex*) analysis of covariance of change scores with baseline scores as a covariate
